# Predictive value of cyst/tumor volume ratio of pituitary adenoma for tumor cell proliferation

**DOI:** 10.1186/s12880-024-01246-z

**Published:** 2024-03-21

**Authors:** Jianwu Wu, Fangfang Zhang, Yinxing Huang, Liangfeng Wei, Tao Mei, Shousen Wang, Zihuan Zeng, Wei Wang

**Affiliations:** 1 Department of Neurosurgery, 900 Hospital of the Joint Logistics Team, No. 156 Xi’erhuanbei Road, Fuzhou, 350025 P. R. China; 2 Department of Endocrinology, the Affiliated Fuzhou First Hospital of Fujian Medical University, Fuzhou, 350009 P. R. China; 3 Department of Neurosurgery, Shenzhen University General Hospital, Shenzhen, 518000 P. R. China; 4 Department of Neurosurgery, the First Affiliated Hospital of Wenzhou Medical University, No. 2, Fuxue Lane, Wuma Street, Lucheng District, Wenzhou, 325000 P. R. China

**Keywords:** Cyst/tumor volume ratio, Proliferation activity, Predictive value, Pituitary adenoma

## Abstract

**Background:**

MRI has been widely used to predict the preoperative proliferative potential of pituitary adenoma (PA). However, the relationship between the cyst/tumor volume ratio (C/T ratio) and the proliferative potential of PA has not been reported. Herein, we determined the predictive value of the C/T ratio of PA for tumor cell proliferation.

**Methods:**

The clinical data of 72 patients with PA and cystic change on MRI were retrospectively analyzed. PA volume, cyst volume, and C/T ratio were calculated. The corresponding intraoperative specimens were collected. Immunohistochemistry and hematoxylin–eosin staining were performed to evaluate the Ki67 index and nuclear atypia. Patients were categorized according to the Ki67 index (< 3% and ≥ 3%) and nuclear atypia (absence and presence). Univariate and multivariate analyses were used to identify the significant predictors of the Ki67 index and nuclear atypia. The receiver operating characteristic curve assessed the prediction ability of the significant predictors.

**Results:**

Larger tumor volumes, smaller cyst volumes, and lower C/T ratios were found in patients with higher Ki67 indexes and those with nuclear atypia (*P* < 0.05). C/T ratio was an independent predictor of the Ki67 index (odds ratio = 0.010, 95% confidence interval = 0.000–0.462) and nuclear atypia (odds ratio = 0.010, 95% confidence interval = 0.000–0.250). The predictive value of the C/T ratio did not differ significantly from that of tumor volume (*P* > 0.05) but was better than that of cyst volume (*P* < 0.05). The area under the curve of the C/T ratio for predicting the Ki67 index and nuclear atypia was larger than that for predicting cyst volume and tumor volume.

**Conclusions:**

C/T ratios can be used to predict PA tumor proliferation preoperatively. Our findings may facilitate the selection of surgery timing and the efficacy evaluation of surgery.

## Background

Pituitary adenomas (PAs) are benign intracranial tumors. However, some PAs contain highly proliferative cells and their volumes may increase, thus invading and compressing the surrounding structures of the sellar region, and resulting in blindness or abnormalities in hormone levels [1, 2]. Early surgical resection of PAs is recommended to decompress pressure in the sellar region, protect the optic nerve, and maintain pituitary function. There may be cystic changes in PAs due to hemorrhage and necrosis [3, 4]. However, the disappearance of the PA has been reported in patients with cystic changes [5, 6], especially in those with no clinical symptoms. Therefore, accurate preoperative prediction of cell proliferation in PA is necessary for the selection of treatment and timing of surgery.

PAs have been traditionally evaluated with the Knosp grading, which only reflects the invasiveness of PA and indirectly reflects the degree of cell proliferation [7, 8]. However, it cannot directly reveal the proliferation rate and thus has limited reliability. PA volume is often used to evaluate and monitor the efficacy of surgery or drug treatment [9], however, the effect of tumor hemorrhage and necrosis on cell proliferation in PA is often ignored. Therefore, efforts are needed to improve the accuracy of preoperative prediction, therefore facilitating decisions related to treatment implementation.

Ki67 is a nuclear protein expressed in proliferating cells, and it is currently considered the gold standard for evaluating PA proliferation [10, 11]. Furthermore, nuclear atypia, such as different nuclear sizes, pleomorphism, and excessive staining, is characteristic of the tumor cells [12]. Magnetic resonance imaging (MRI) has been widely used to predict the preoperative proliferative potential of PA. For example, the MIB-1 labeling index has been reported to be correlated with tumor volume doubling time in PA [13]. Preoperative T2 MRI texture analysis is effective in predicting the Ki-67 proliferation index of PA [14]. However, to the best of our knowledge, the relationship between the cyst/tumor volume ratio (C/T ratio) and proliferative potential of PA has not been reported.

Herein, we analyzed the relationship of the C/T ratio with the Ki67 index and nuclear atypia and explored whether the C/T ratio can help predict PA cell proliferation. Our findings may improve the accuracy of preoperative prediction of PA tumor proliferation.

## Methods

### Participants

This is a retrospective study. Patients with PA who were examined in the Neurosurgery Department of our hospital between January 2018 and September 2019 and whose MRI showed cystic changes in the tumor were recruited. Inclusion criteria: 1) patients underwent preoperative MRI and surgical treatment; 2) patients with PA diagnosed according to postoperative pathological findings; and, 3) cystic changes of PA were observed on T2-weighted images (T2WIs) with a maximum diameter ≥ 2 mm. Exclusion criteria: 1) patients did not receive surgical treatment for PA; 2) patients with a previous history of surgery, radiotherapy, or drug therapy; and, 3) Patients with other lesions in the sellar region. PA tissues were collected during surgery. The study was performed following the declaration of Helsinki. This study was approved by the Ethics Committee of the 900 Hospital of the Joint Logistics Team (No. 2021–005). Written informed consent was waived by the Ethics Committee of the 900 Hospital of the Joint Logistics Team, as the clinical data were anonymized and retrospectively analyzed.

### Data collection

The baseline data of the study participants were collected, such as age, gender, tumor volume, cyst volume, Ki67 index, and nuclear atypia.

### MRI

All the enrolled patients underwent plain and enhanced MRI of the pituitary gland with the 3.0-T MRI scanner (Siemens Healthcare, Erlangen, Germany) within 1 week before surgery. A 24-channel head/neck coil was used. The coronal, sagittal, and three-dimensional (3D) enhanced T1-weighted images (T1WIs) and T2-weighted images (T2WIs) were obtained. The imaging protocol included pre-contrast and contrast-enhanced coronal T1 sequences (repetition time (TR)/echo time (TE), 400/6.7 ms; slice thickness, 2 mm; 12 sections; field of view (FOV), 200 mm; flip angle, 150°; bandwidth, 399 Hz/pixel; pixel resolution, 256), contrast-enhanced sagittal T1 sequences (TR/TE, 440/3.5 ms; slice thickness, 2 mm; 12 sections; FOV, 240 mm; flip angle, 150°; bandwidth, 380 Hz/pixel; pixel resolution, 320), coronal T2 sequences (TR/TE, 3000/103 ms; slice thickness, 2 mm; 12 sections; FOV, 200 mm; flip angle, 150°; bandwidth, 260 Hz/pixel; pixel resolution, 320), and sagittal T2 sequences (TR/TE, 4000/110 ms; slice thickness, 2 mm; 12 sections; FOV, 260 mm; flip angle, 150°; bandwidth, 223 Hz/pixel; pixel resolution, 320). Gadopentetate dimeglumine or gadobenate dimeglumine was used as the contrast agent, and the dose was 0.2 mL/kg of body weight. T2WIs were obtained before the injection of the contrast agent.

### Image analysis

**Fig. 1:**
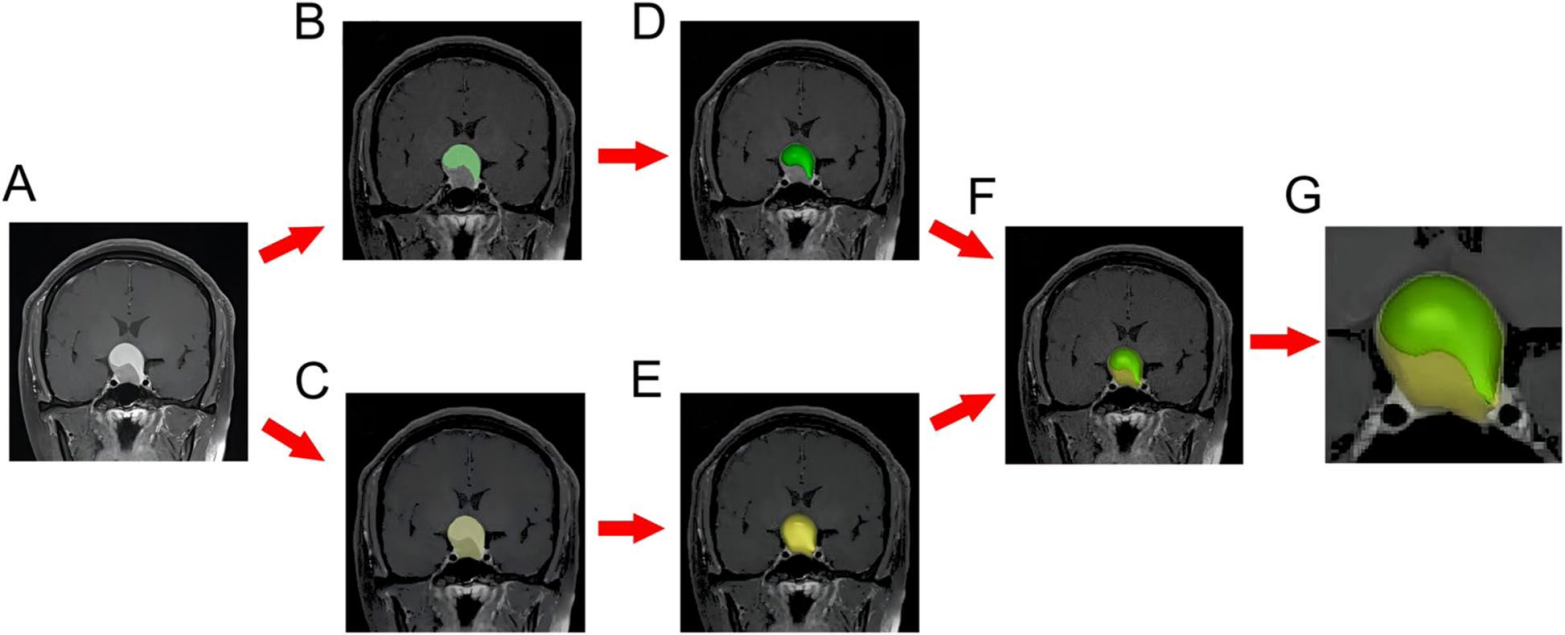
Method for measuring tumor volume and cyst volume using the Mimics software. **A** The original images with a clear presentation of the tumors and cysts were selected. **B** and **C** The original images were imported into Mimics software, and the “Thresholding” and “Edit Masks” tools were used to distinguish the dimensions of the tumors and cysts. **D** and **E** Three-dimensional (3D) reconstruction of the cysts was performed with the “Calculate 3D” tool. **F** and **G** The position relationship between the cyst and tumor was identified by combining the reconstructed 3D masks. After making them transparent, the volume of the tumor (yellow) and cyst (green) were directly read through the “Properties” option

MRI data were processed using the INFINITT PACS Medical Imaging System (syngo. via software VB10; Siemens Healthcare, Germany). Preoperative MRI data were evaluated by two experienced neurosurgeons (S.W., with 34 years of experience, and L.W., with 25 years of experience). Evaluations were made based on the presence of cystic change on MRI and using the Knosp grade [15]. To calculate the C/T ratio, we imported the obtained data in DICOM format into Materialise Mimics 19.0 software (Materialise, Kanagawa, Japan). The images were processed according to the following procedures: 1) The original images with a clear presentation of tumors and cysts were selected (Fig. 1A) and the threshold segmentation of the tumor structure was performed by using the “Thresholding” tool of the software. 2) The regions were expanded with the “Region Growing” tool and the images were saved as masks. 3) The masks were edited with the “Edit Masks” tool to isolate the brain tissue, cavernous sinus, skull base, and other structures that connected the tumor and cyst, slice by slice (Fig. 1B and C). 4) The 3D reconstruction of the tumors or cysts was conducted with the “Calculate 3D” tool, based on the masks (Fig. 1D and E). 5) The small holes were filled by using the “Morphology operations” tool and the tumor surfaces were smoothed with the “Smoothing” tool (Fig. 1F and G). 6) The tumor and cyst volumes were read directly using the “Properties” option.

### Immunohistochemistry

The PA tissues were fixed, dehydrated, paraffin-embedded, and cut into 4-μm sections. Immunohistochemistry was conducted as previously described [16]. Briefly, after the inactivation of endogenous peroxidase, antigen retrieval, and blocking, the sections were incubated with Ki67 antibody (1:200, Abcam) and the corresponding secondary antibody. Finally, the sections were observed under an optical microscope (Model BX-51, Olympus, Tokyo, Japan), and the images were analyzed with Image-Pro Plus 6.0 (Media Cybernetics, Inc., Rockville, MD, USA). The number of cells that were stained positively for Ki67 were counted in 5 random fields under × 200 magnification. A Ki67-positive percentage of ≥ 3% indicated positive staining, whereas < 3% indicated negative staining [17].

### Hematoxylin and eosin staining

PA specimens were prepared into sections as above described, and then they were stained with hematoxylin and eosin according to the previous description [18]. The degree and percentage of nuclear atypia were evaluated in 5 high-power (× 200) fields. For the degree of nuclear atypia, 1 point indicated that the nuclei were consistent in size and shape, were not hyperchromatic, had uniform hyperchromatic dispersion of chromatin, or had no fine-grained chromatin condensation; 3 points indicated that the nuclei had different sizes or pleomorphism, were stained excessively, and were rough and irregularly distributed; and, 2 points indicated that the features of nuclear atypia were between those of 1 and 3 points. The percentage of nuclear atypia was scored as follows: 0 points, percentage of < 10%; 1 point, percentage of 10%–25%; 2 points, percentage of 25%–50%; and, 3 points, percentage of > 50%. The final score of nuclear atypia was obtained by multiplying the score of nuclear atypia degree and nuclear atypia percentage. A score of ≥ 3 points indicated the presence of nuclear atypia [19].

### Statistical analysis

SPSS 23.0 (SPSS Inc., Chicago, IL, USA) was used to statistically analyze all data. Continuous variables are described by medians with interquartile ranges (the first and third quartiles), and categorical variables are presented as percentages. The Mann–Whitney *U* test was used to compare the Ki67 indexes (< 3% and ≥ 3%) and nuclear atypia (absence and presence). Factors that were statistically different (*P* < 0.05) were included in the multivariate binary Logistic regression to determine the independent predictors of Ki67 and nuclear atypia. MedCalc software (version 18.2) was used to plot the receiver operating characteristic curve. The area under the curve (AUC) was calculated. *P* value < 0.05 was considered statistically significant.

## Results

### Basic characteristics of patients

**Fig. 2:**
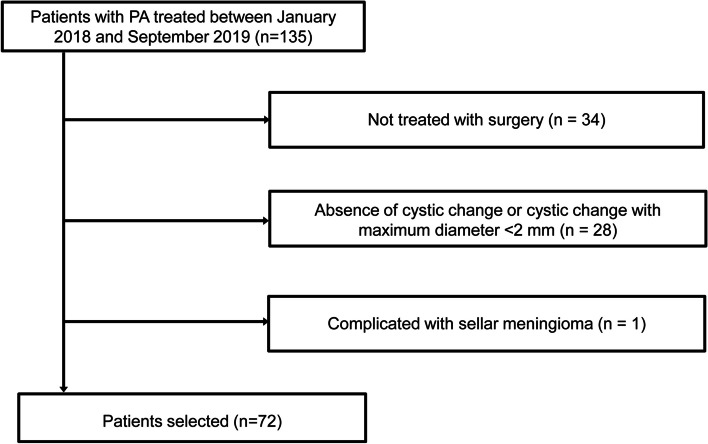
Flowchart of patient enrollment. PA, pituitary adenoma

**Fig. 3:**
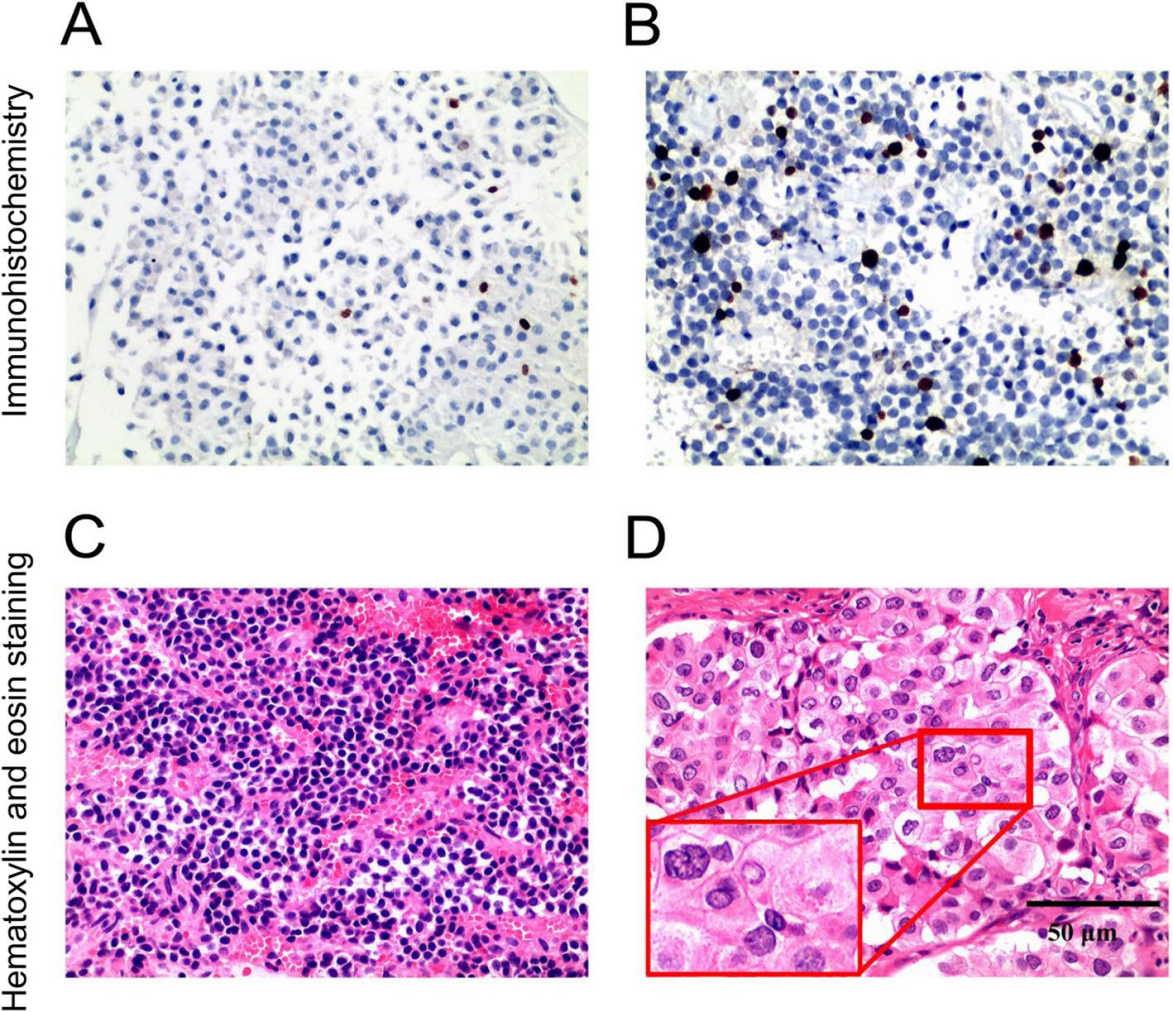
Immunohistochemistry analysis of Ki67 and hematoxylin and eosin staining. **A** The representative image with a Ki67 index of < 3%. Most nuclei are blue-cyan, and a few are dark brown. **B** The representative image with a Ki67 index of ≥ 3%. The proportion of dark brown nuclei (blue arrow) is ≥ 3%. **C** The representative image with the absence of nuclear atypia. The nuclei have regular shapes and are not enlarged. **D** The representative image with the presence of nuclear atypia. The nuclei have irregular and inconsistent shapes and enlarged volume (blue arrow). Magnification: × 200. Scale bar: 50 μm

Figure 2 is the flowchart of patient enrollment. Of the 135 patients with PA who were potentially eligible for this study, 63 patients were excluded, including 34 patients who were not treated with surgery, 28 patients who had no cystic change or had cystic change with maximum diameter < 2 mm on T2WI, and 1 patient who was complicated with sellar meningioma. Finally, 72 patients were included. Their basic clinical data is shown in Table 1. Representative immunohistochemistry results of Ki67 and hematoxylin and eosin staining results of nuclear atypia were shown in Fig. 3. The Ki67 expression was positive (≥ 3%) in 25 patients (34.72%), and nuclear atypia (≥ 3 points) was present in 28 (38.89%) patients (Table 1).

**Table 1:** Clinical data of the study participants

Variables	Value
Median age, years (IQR)	53.90 (16.46–81.46)
Number of female patients (%)	32 (42.67)
Median cyst volume (IQR) (cm^3^)	0.366 (0.084–1.796)
Median tumor volume (IQR) (cm^3^)	4.573 (1.849–8.790)
Median C/T ratio (IQR)	0.187 (0.018–0.570)
Number of microadenomas (maximum diameter, < 1 cm)	1 (1.4)
Number of macroadenomas (maximum diameter, 1–3 cm)	63 (87.5)
Number of giant adenomas (maximum diameter, ≥ 4 cm)	8 (11.1)
Knosp grade (*n* [%])	
0	12 (16.7)
1	19 (26.4)
2	13 (18.1)
3	10 (13.9)
4	18 (25.0)
IHC classification (*n* [%])	
ACTH	12 (16.7)
FSH	6 (8.3)
GH	1 (1.4)
LH	25 (34.72)
Multiple hormone type	7 (9.7)
Null	11 (15.2)
PRL	10 (13.9)
Percentage of patients with Ki67 index of ≥ 3% (*n* [%])	25 (34.72)
Percentage of patients with nuclear atypia (*n* [%])	28 (38.89)

*ACTH* adrenocorticotropic hormone, *C/T* the ratio of cyst volume to tumor volume, *FSH* follicle-stimulating hormone, *GH* growth hormone, *IHC* immunohistochemistry, *IQR* interquartile range, *LH* luteinizing hormone, *PRL* prolactin

### Univariate and binary multivariate Logistic analyses of predictors of Ki67 index and nuclear atypia

Univariate analysis of factors associated with the Ki67 index and nuclear atypia showed that age, sex, and Knosp grade did not differ significantly among groups classified by the Ki67 index (< 3% and ≥ 3%) and by nuclear atypia (absence and presence). Compared with patients with Ki67 < 3% and those without nuclear atypia, patients with Ki67 ≥ 3% and those with nuclear atypia had significantly larger tumor volumes (*P*_Ki67_ = 0.001 and *P*_nuclear atypia_ = 0.014), smaller cyst volumes (*P*_Ki67_ = 0.005 and *P*_nuclear atypia_ = 0.038), and lower C/T ratios (*P*_Ki67_ < 0.001 and *P*_nuclear atypia_ = 0.001) (Table 2). The significant factors of tumor volume, cyst volume, and C/T ratio were included in the multivariate binary Logistic analysis, and the results showed that the C/T ratio was an independent predictor of Ki67 index (odds ratio = 0.010, 95% confidence interval (CI) = 0.000–0.462, *P* = 0.006) and nuclear atypia (odds ratio = 0.010, 95% CI = 0.000–0.250, *P* = 0.005) (Table 3).

**Table 2:** Univariate analysis of factors associated with Ki67 index and nuclear atypia

	**Ki67 index**			**Nuclear atypia**		
**Variables**	** < 3% (*n* = 47)**	** ≥ 3% (*n* = 25)**	***P* value**	**Absent (*n* = 44)**	**Present (*n* = 28)**	***P* value**
Median age, years (IQR)	55.83 (44.92–63.45)	53.91 (46.22–62.28)	0.845	55.70 (47.10–66.10)	54.87 (44.58–61.89)	0.439
Number of male patients (%)	22 (46.8%)	10 (40.0%)	0.580	22 (50.0%)	10 (35.7%)	0.234
Median cyst volume (IQR) (cm^3^)	0.98 (0.14–2.89)	0.10 (0.04–0.32)	0.005^*^	0.87 (0.15–2.51)	0.10 (0.05–0.99)	0.038^*^
Median tumor volume (IQR) (cm^3^)	4.12 (0.99–5.35)	8.90 (3.41–12.07)	0.001^*^	4.12 (1.12–6.65)	6.63 (2.35–12.39)	0.014^*^
Median C/T ratio (IQR)	0.36 (0.11–0.70)	0.02 (0.01–0.11)	< 0.001^**^	0.36 (0.04–0.71)	0.02 (0.01–0.16)	0.001^*^
Knosp grade (*n* [%])			0.611			0.858
0	9 (19.1)	3 (12.0)		7 (15.9)	5 (17.9)	
1	14 (29.8)	5 (20.0)		13 (29.5)	6 (21.4)	
2	7 (14.9)	6 (24.0)		7 (15.9)	6 (21.4)	
3	7 (14.9)	3 (12.0)		7 (15.9)	3 (10.7)	
4	10 (21.3)	8 (32.0)		10 (22.7)	8 (28.6)	

*C/T* cyst/tumor volume ratio, *IQR* interquartile range

^*^P < 0.05; ^**^P < 0.001

**Table 3:** Binary multivariate Logistic analysis of predictors of Ki67 index and nuclear atypia

Variable	Regression coefficient (*β*)	Standard error	*P* value	OR	95% CI
Ki67 index (≥ 3%)					
Cyst volume	− 0.048	0.221	0.921	0.821	0.631–1.441
Tumor volume	0.044	0.047	0.396	1.045	0.953–1.146
C/T ratio	− 4.601	1.954	0.006^*^	0.010	0.000–0.462
Nuclear atypia (presence)					
Cyst volume	0.261	0.211	0.217	1.298	0.858–1.964
Tumor volume	0.011	0.103	0.748	1.011	0.947–1.078
C/T ratio	− 4.609	7.856	0.005^*^	0.010	0.000–0.250

*C/T* ratio of cyst volume to tumor volume, *CI* confidence interval, *OR* odds ratio

^*^P < 0.05

### Receiver operating characteristic curve analysis of cyst volume, tumor volume, and C/T ratio in predicting Ki67 index and nuclear atypia

**Fig. 4:**
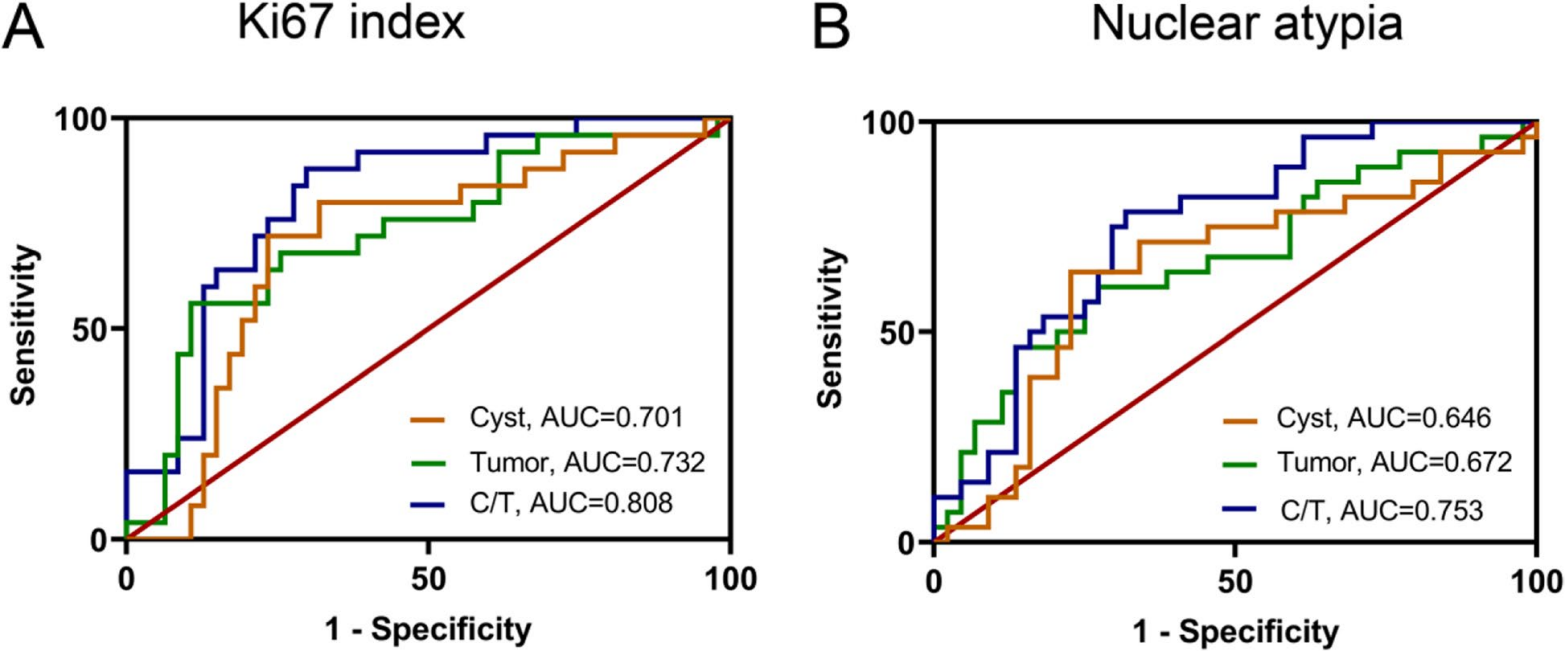
Receiver operating characteristic curves of Ki67 index and nuclear atypia.** A** Ki67 index. **B** Nuclear atypia. AUC, the area under the curve; C/T, the ratio of cyst volume to tumor volume

The receiver operating characteristic curve analysis demonstrated that for the prediction of the Ki67 index, the cutoff value of cyst volume was 0.188 (Table 4). The AUC of cyst volume was 0.701, with a sensitivity of 72.00% and a specificity of 74.47% (Fig. 4A and Table 4). For the prediction of nuclear atypia, the cutoff value of cyst volume was 0.148 (Table 4). The AUC of cyst volume was 0.646, with a sensitivity of 64.29% and a specificity of 75.00% (Fig. 4B and Table 4). For the prediction of the Ki67 index, tumor volume had an AUC of 0.732, a cutoff value of 8.13, a sensitivity of 89.7%, and a specificity of 86.8% (Fig. 4A and Table 4). For the prediction of nuclear atypia, tumor volume had an AUC of 0.672, a threshold of 5.184, a sensitivity of 89.7%, and a specificity of 86.8% (Fig. 4B and Table 4). For the C/T ratio, its cutoff value for prediction of the Ki67 index was 0.159, and its AUC was 0.808, with a sensitivity of 89.7% and a specificity of 86.8% (Fig. 4A and Table 4). For the prediction of nuclear atypia, the cutoff value of the C/T ratio was 0.159, with an AUC of 0.753, a sensitivity of 89.7%, and a specificity of 86.8% (Fig. 4B and Table 4). The AUC of the C/T ratio for predicting either the Ki67 index or nuclear atypia was larger than those of cyst volume and tumor volume.

**Table 4:** Receiver operating characteristic curve analysis of cyst volume, tumor volume, and C/T value in predicting Ki67 index and nuclear atypia

Parameter	Cutoff value	AUC	95% CI	Sensitivity (%)	Specificity (%)	Youden index (%)	*P* value
Ki67 index (≥ 3%)							
Cyst volume	0.188	0.701	0.576–0.833	72.00	74.47	0.467	0.004^*^
Tumor volume	8.13	0.732	0.607–0.857	56.00	89.36	0.454	0.001^*^
C/T ratio	0.159	0.808	0.710–0.914	88.00	68.09	0.561	< 0.001^**^
Nuclear atypia (presence)							
Cyst volume	0.148	0.646	0.513–0.786	64.29	75.00	0.392	0.034^*^
Tumor volume	5.184	0.672	0.541–0.803	60.71	72.73	0.334	0.014^*^
C/T ratio	0.159	0.743	0.637–0.862	78.57	65.91	0.445	< 0.001^**^

*AUC* area under the curve, *C/T* ratio of cyst volume to tumor volume

^*^ *P* < 0.05; ^**^P < 0.001

*Z* tests revealed no statistical difference between C/T ratio and tumor volume (*Z*_Ki67_ = 1.001, *P*_Ki67_ = 0.136; *Z*_nuclear atypia_ = 0.237, *P*_nuclear atypia_ = 0.813) or between tumor volume and cyst volume (*Z*_Ki67_ = 0.297, *P*_Ki67_ = 0.767; *Z*_nuclear atypia_ = 0.891, *P*_nuclear atypia_ = 0.373) in predicting Ki67 index and nuclear atypia, but C/T ratio had a higher predictive value than cyst volume (*Z*_Ki67_ = 2.975, *P*_Ki67_ = 0.003; *Z*_nuclear atypia_ = 2.469, *P*_nuclear atypia_ = 0.014).

## Discussion

Although the association of tumor size with PA cell proliferation has been reported previously [13, 20, 21], this report is, to the best of our knowledge, the first to propose the concept of C/T ratio and to demonstrate the value of this ratio in predicting PA cell proliferation. This calculation of the C/T ratio was simple and easy, which only needed MRI data. Based on this ratio, a preliminary prediction about the progression of PA could be made, and thus the treatment could be planned at an earlier time.

Tumor volume is associated with the proliferation and malignancy of tumor cells and, in clinical practice, is an important factor affecting the survival of patients and prognosis [22–25]. However, the evaluation of PA cell proliferation based on PA volume cannot account for the effect of hemorrhage or necrosis on the growth of PAs. When the amount of PA hemorrhage or necrosis exceeds the rate of cell proliferation, the growth of the tumor slows down, and the tumor may even disappear on its own [5, 26]. Cavalli et al. reported that tumors tended to disappear in patients with intratumoral hemorrhage and cystic change on preoperative MRI [27]. Therefore, the C/T ratio in this study, when used for the evaluation of PA cell proliferation, could comprehensively account for the contradiction between PA necrosis and proliferation, and thus may help improve the accuracy of preoperative prediction.

PA volume is often calculated according to the Coniglobus formula (volume = π/6 × length × width × height). However, the Coniglobus formula is suitable for objects that have regular shapes of the approximate ellipse, but it is not suitable for tumors or hematomas that have irregular shapes, in which the calculation error can be large [28, 29]. PAs that invade surrounding structures of the sellar region usually have irregular shapes and volumes [30, 31]. Moreover, when used for calculating tumor volume, the Coniglobus formula is based on two-dimensional data, and thus the selection of images at different levels will also cause changes in the calculation results [32]. For this reason, we used 3D reconstruction in this study to assess tumor and cyst volumes. The Mimics software not only can measure tumors or cysts with irregular shapes but also has higher evaluation accuracy than traditional calculation methods [33].

Ki67 is a proliferation antigen expressed in nuclei, and its levels can reflect the proliferation of tumor cells. However, recent studies have shown that Knosp grading cannot accurately predict the Ki67 index before surgery [34–36]. This is because the measurement method in Knosp grading accounts for only the invasiveness of PA into the cavernous sinus, however, it does not account for the effects of other areas in the sellar region. Therefore, PA invasiveness cannot fully reflect PA cell proliferation. Moreover, the Knosp grading represents only two-dimensional data but not the 3D spatial structure of the tumor. The proliferation of PA cells is associated with both the Ki67 index and nuclear morphology. Popescu et al. indicated that highly proliferative PA cells had pleomorphism, a high nuclear-cytoplasmic ratio, and clear nucleoli, whereas the cells of non-proliferative PAs had more consistent nuclear morphology, a low nuclear-cytoplasmic ratio, and unclear nucleoli [37]. In our study, the Ki67 index and nuclear atypia were selected as the pathological indicators to evaluate the proliferation of PA cells. We found larger tumor volumes, smaller cyst volumes, and lower C/T ratios in patients with Ki67 of ≥ 3% and those with nuclear atypia than in patients with Ki67 of < 3% and in those without nuclear atypia. This finding suggests that cystic change may have an inhibitory effect on the proliferation of PA cells. For the prediction of the Ki67 index and nuclear atypia, the ability of the C/T ratio did not differ significantly from that of the tumor volume but was significantly better than that of the cyst volume. The AUC of the C/T ratio for predicting the Ki67 index and nuclear atypia was higher than those of the tumor volume and cyst volume. Logistic regression showed that the C/T ratio was an independent predictor of the Ki67 index and nuclear atypia. These results suggest that the C/T ratio has a relatively high accuracy in the preoperative prediction of PA cell proliferation.

This study has some limitations. For example, the sample size was relatively small. Thus, we could not analyze the data based on the 2017 version of the pathological classification of PA [38], and only performed immunohistochemical staining. Moreover, the dynamic post-contrast evaluation was not performed. Another study limitation is the vast difference between median cyst and tumor volumes and, consequently, the low median C/T ratio. Further studies are warranted.

## Conclusions

Our findings suggest that the C/T ratio calculated with preoperative MRI data can be used to predict the cell proliferation of PA. It has a higher accuracy than Knosp grading, tumor volume, and cyst volume. With the 3D reconstruction software, the C/T ratio can be calculated easily and accurately. Preoperative prediction of PA cell proliferation by the C/T ratio may facilitate the selection of surgery timing and the efficacy evaluation of surgery.

## Data Availability

The datasets used and/or analyzed during the current study are available from the corresponding author upon reasonable request.

## Abbreviations

AUC: Area under the curve
C/T: Cyst/tumor volume ratio
MRI: Magnetic resonance imaging
PA: Pituitary adenoma
